# Explaining external economic support inequality among households affected by HIV/AIDS in Tanzania: an Oaxaca Blinder decomposition analysis

**DOI:** 10.1186/s13561-022-00363-1

**Published:** 2022-03-04

**Authors:** Wilfried Guets, Edward Kwabena Ameyaw, Sanni Yaya

**Affiliations:** 1 Health, Nutrition, and Population Unit, The World Bank, Washington, DC USA; 2 The Australian Centre for Public and Population Health Research (ACPPHR), Faculty of Health, University of Technology Sydney, Ultimo, Australia; 3 School of International Development and Global Studies, University of Ottawa, Ottawa, Canada; 4 The George Institute for Global Health, Imperial College London, London, UK

**Keywords:** HIV/AIDS, Economic support, Oaxaca-Blinder decomposition, Households, Tanzania

## Abstract

**Background:**

HIV/AIDS remains the leading cause of death in sub-Saharan Africa. Due to multiple constraints experienced by households that seem to be disproportionally affected, families generally seek assistance from the community and external economic support. Previous researchers studied socioeconomic and gender inequality in HIV/AIDS prevalence in sub-Saharan African countries. However, very few researchers have paid attention to the external economic support for HIV/AIDS affected households in Tanzania. This study investigates the difference in economic support among households affected or not affected by the HIV/AIDS epidemic in Tanzania.

**Methods:**

Data used stemmed from the Tanzania HIV Impact Survey 2016–2017 (THIS) of the Population-based HIV Impact Assessment (PHIA) project, collected between 2016 and 2017 in Tanzania. The study population were the heads of households (adults) with age greater than 15. The dependent variable for the study was economic support. This consisted of both material and non-material assistance obtained from outside the household. Socio-demographic (economics) characteristics constituted the predictors of the study. Descriptive statistics and econometric modelling were used to analyse determinants associated with external economic support. Oaxaca-Blinder decomposition method was also performed to investigate the difference in economic support depending on households’ serological status in Tanzania.

**Results:**

A total of 12,008 households were included. Almost 11% of the household heads indicated that their households received economic support. HIV/AIDS affected 7% of households. The mean age of the household heads was 45 years (SD ± 15) with a range of 16–80. The majority of household heads were men (72%). Being a household head affected by HIV/AIDS increases the probability to receive external economic support (*p* < 0.05). The difference in external economic support between the two groups (HIV/AIDS and no- HIV/AIDS households) was - 0.032 (*p* < 0.01). This gap was observed to favour households affected by HIV/AIDS. Almost 72% (− 0.023/− 0.032) of this difference was explained by characteristics such as the wealth index (*p* < 0.01), residence area (urban) (*p* < 0.01), marital status (widowed (*p* < 0.05) and divorced or separated) (*p* < 0.1) and age (*p* < 0.01).

**Conclusion:**

The difference in economic support across households affected or not affected by HIV/AIDS was explained by wealth index, residence area, marital status and age. These findings represent important implications for health policy regarding future economic support strategies for HIV/AIDS-affected households.

## Background

Globally, over 38.0 million persons are living with HIV/AIDS comprising 36.2 million adults and 1.8 million children (0–14 years). In 2019, 69% of new HIV infections were reported from western and central Africa [1], whilst one million people get infected each year globally (with 84% younger than 50 years old) [2]. HIV/AIDS remains the leading cause of death in sub-Saharan Africa [3]. Developing countries are challenged with several ill-health conditions such as infectious and parasitic diseases (malaria, tuberculosis) and respiratory infections, maternal and neonatal conditions, and other existing non-communicable diseases. In sub-Saharan African countries, the population is very concerned about HIV/AIDS, particularly in South Africa and Nigeria, where the is a high prevalence [4].

In Tanzania, HIV/AIDS affects 1.33 million persons, and it is clear that this pandemic remains a predominant health issue. Therefore, infected people can sometimes experience challenges, such as stigma and depression [5]. Different health programs were implemented and conducted at the local level. However, the HIV/AIDS epidemic affects the entire economy, particularly the health sector. As a result, HIV/AIDS epidemic’s socioeconomic consequences can be observed at the community level (household) and the macroeconomic level [6–9].

One component directly impacted by HIV/AIDS at the macroeconomic level is the economic growth rate [10]. Some research regarding the macroeconomic outlooks in sub-Saharan countries indicated that the epidemic would slow economic growth [11, 12]. However, the prevision could depend on multiple assumptions, such as the number of people affected and the household contribution (saving) dedicated to health care. A study of the growth trajectories conducted in 1992 for 30 sub-Saharan countries over the period 1990–2025 concluded that economic growth rates would be reduced between 0.56 and 1.47% [13]. In 2000, Bonnel indicated that HIV/AIDS lowered the growth rate of Africa’s per capita GDP by 0.7% per year from 1990 to 1997. This growth was reduced by a further 0.3% per year when countries were affected by the malaria epidemic [14]. Other studies also shed light on how HIV/AIDS reduced economic growth by destroying human capital (young adults), diminishing and slowing down the mechanism generating the human capital and investment in people [15, 16]. The socioeconomic impact of HIV/AIDS could lead to the loss of jobs, income, and death of family members, such as parents reduced education and knowledge of the new generation, reduced interest in being well educated and then, invest less in health [17, 18]. Considering that the macroeconomic growth is compromised, this will induce significant implications for government level spending [10]. The health and social care services domains may be severely affected due to the inadequately use of the health workforce, a need for additional support (fund) [6], and an increase in long term health spending in some emerging economies [19].

At the national level, the impact of HIV/AIDS is devastating to the community, family and household. Even though the consequences of the epidemic on households were quite noticeable, early research failed to capture affected households and needing economic support. According to [20, 21], households’ impacts appear when a family member of the household starts to suffer from the burden associated with HIV/AIDS, such as loss of patient income and substantial household expenditure on medical expenses. Other family members (e.g., daughters and partners) may miss school or work to assist the person in need. Besides, it results in a sustainable loss of productivity (income) from less labour on the farm or lower remittances, bereavement costs (funeral), and children dropping out of school [9]. Other research shows that HIV/AIDS can also affect child development [22] and the vulnerability of families [23]. Some programs have been implemented to target and support households affected by HIV/AIDS in sub-Saharan Africa. Some bilateral partners and funders of many countries (e.g. UNAIDS and USAID) have provided a variety of projects to progress towards the achievement of the Sustainable Development Goals (SDGs), in particular, the target 3.3 [24, 25]. Tanzania has adopted a global plan for the elimination of HIV infection among children born to HIV-infected mothers and for keeping their mothers alive (eMTC, 2012–2015); a Prevention of Mother-to-Child Transmission (PMTCT) Programme (2016) and the 2017 Zanzibar Integrated HIV, Hepatitis, Tuberculosis and Leprosy, Programme (ZIHHTLP).

Community play an essential role in support of the family members. Due to multiple constraints experienced by HIV/AIDS affected households, the families generally seek assistance from the community level (friends, close acquaintances) and external economic support. Therefore, to reduce the burden of HIV/AIDS, the household should maintain the internal income by benefiting from the resources and contribution of family members. Very few research have paid attention to the external economic support regarding HIV/AIDS affected households. Previous analyses studied socioeconomic and gender inequality in HIV/AIDS prevalence in sub-Saharan African countries [26–28]. This study investigates the gap in external economic support between households with HIV/AIDS and those without HIV/AIDS using a non-linear Oaxaca-Blinder decomposition. This paper represents a significant contribution to the literature because it constitutes the first research mobilising the large and recent nationally representative data set collected in Tanzania.

## Method and data

### Data source

We used data from the Tanzania HIV Impact Survey 2016–2017 (*THIS*) of the Population-based HIV impact assessment (PHIA) project, collected between November 2016 and June 2017 in Tanzania [4]. *THIS* is a cross-sectional and national representative, population-based survey aiming to provide information on HIV/AIDS indicators on the population-level impact of HIV-related prevention, care, and treatment interventions.^1^ Participants were the heads of households (adults) with an age greater than 15 years old.^2^ A face-to-face interview was used across 31 regions of the country.

The subnational geographic (region) strata implemented a stratified multistage survey sampling design. The data collection included three steps. In the first step, census enumeration areas (EAs) were randomly selected with a population size proportion probability. In the second step, a sample was randomly selected based on the households selected in the first step. Individual questionnaires were administered to eligible and consenting individuals in the household. In the third step, children under 15 years were targeted among a subset of selected households. Households and individual (adults: 15 + years) surveys questionnaire contained several modules: household characteristics, HIV knowledge, marriage, alcohol use, and particularly economic support.

### Ethical consideration

Protocol for the THIS was reviewed and approved by the institutional review boards of CDC, Columbia University, Westat, the National Institute for Medical Research, and Zanzibar Medical Research and Ethics Committee before data collection [4]. All enumerators and survey staff, such as laboratory technologists, nurse interviewers, and supervisors, were trained on good clinical and laboratory practices as well as ethical protection of survey respondents, and each signed a data confidentiality agreement [4].

### Variables

Individuals and/or households questionnaires presented different categories of variables per module. The economic support module contains multiple variables related to support. Regarding the economic support (dependent variable), participants were asked to answer the question: *Has your household received any of the following forms of external economic support in the last 12 months?* The households were likely to benefit from a range of external economic support. The various support provided at the household level contained: cash transfer (e.g., pensions, disability grants, child grants); assistance for school fees; material support for education (e.g., uniform, schoolbooks, etc.), income generation support in cash or in-kind combination of any; food assistance provided at the household or external institution; material or financial support for shelter; social pension, and other. The distribution of economic support among support was not the same among households. Some individuals were likely to benefit from more than one type of support compared to others. Economic support was defined as “1” if the household benefits from any form of support and “0” if the household head did not receive economic support.

The HIV/AIDS serostatus was determined by pre-specified HIV testing algorithms that generally included a combination between home-based rapid HIV and confirmation with laboratory-based testing. Only two finals results of HIV/AIDS tests were possible: “positive” for infected persons and “negative” for non-infected persons. We considered these two groups for our analysis. It is worth noting that some respondents were not informed of their serostatus (not tested or had no definite outcome). As in previous studies, we used the HIV/AIDS serostatus as a binary variable where “1” for positive and “0” for negative.

Socioeconomic and demographic households characteristics were collected. Our analysis is based on a large list of explanatory variables primarily used in the literature [29, 30]. The wealth index measures the level of impoverishment and socioeconomic status of households. This wealth measure is built following the approach adopted by the Demographic and Health Survey (DHS). In practice, the wealth index measurement includes household assets, material and durable goods in the respondent’s house. The wealth index was indicated as a continuous and/or categorical variable. According to the previous studies, households characteristics such as marital status (categorical variable defined by 1 “Married”, 2 “Living together”, 3 “Widowed” and 4 “Divorced or Separated”, education (categorical variable defined by 1 “No education”, 2 “Primary”, 3 “Secondary”; 4 “More than secondary”), gender (binary variable defined by 1 “Female”, 0 “Male”), residence area (binary variable 1 “Urban”, 0 “Rural”), number of children, and age were also used as other explanatory variables to explain the benefit from economic support.

## Methods

Descriptive statistics were used to provide details on the study sample. We then used multivariate logit models to investigate characteristics and factors associated with households’ external economic support. The following model was used:

$$ {EconomicSupport}_i={\beta}_0+{\beta}_1{HIVStatus}_i+{\beta}_2{Wealth}_i+{\beta}_3{Gender}_i+{\beta}_4{Urban}_i+{\beta}_5{NumberChild}_i+{\beta}_6{Education}_i+{\beta}_7{MaritalStatus}_i+{\beta}_8{age}_i+ REGION+{\varepsilon}_i $$

1 $$ i=1,2,\dots, N $$

Where, *EconomicSupport*_*i*_ is the dependent dichotomous variable with a value “1” (i.e. the household received any economic support) or “0” (i.e. the household did not receive economic support). *β*_0_ is a constant and *β*_1_, *β*_3_, …, *β*_8_ represent the coefficient corresponding to explanatory variables to estimate; *HIVStatus*, *Wealth*, *Gender*, *Urban*, *NumberChild*, *Education*, *MaritalStatus* and *age* represent independent variables. Regional specific effects (*REGION*) were included to capture the specificity of all 31 regions of the country in the model. *ε*_*i*_ stands for the error term. In models 1 and 2, the dependent variable was economic support. In models 3, 4 and 5, we used as dependent variables three different forms of economic support particularly: “Cash transfer (e.g. pensions, disability grants, child grants)”; “Assistance for school fees”; “Material support for education (e.g. uniforms, school books, education, tuition support, bursaries)”.

### Oaxaca Blinder decomposition

In the literature, it has been shown that the impact of HIV/AIDS is likely to differ between affected and not affected households [31–33]. The difference in households’ economic support in both HIV/AIDS groups (negative vs positive) was investigated. Some studies used various approaches to measure and capture inequality among the Oaxaca-Blinder decomposition technique [34]. This method explains the difference in the mean of a dependent variable between two groups by decomposing the gap into three components. This decomposition technique was initially used to explain the wage differential between two different groups of workers, particularly by gender or race. This method was used in other research topics, but not many studies applied this technique to address the health economics research question [35]. This method decomposes the difference between three different components: endowments (E), coefficients (C) and interaction (CE) [34, 36]. In brief, the endowment or explained component refers to the difference in determinants or socio-demographic (economics) characteristics. The coefficient or unexplained component refers to coefficients or parameters. The non-linear decomposition methodology is developed in the following framework:

Let us consider the following linear regression model, which is adjusted separately for the groups *g* = (*A*, *B*). For simplification in the reading, categories A and B respectively refer to households with positive and negative serological status:

2 $$ {Y}_{ig}={X}_{ig}\ {\beta}_g+{\varepsilon}_{ig} $$

Where *i* = 1, 2, …, *N*_*g*_ and ∑_*g*_*N*_*g*_ = *N*; *Y*_*ig*_ a continuous dependent variable; *X*_*ig*_ is the vector of explanatory variables [37, 38]. initially developed the following decomposition:

3 $$ {\Delta }^{OLS}={\overline{Y}}_A-{\overline{Y}}_B=\left({\overline{X}}_A-{\overline{X}}_B\right){\hat{\beta}}_A+{\overline{X}}_B\left({\hat{\beta}}_A-{\hat{\beta}}_B\right) $$

Where ${\overline{Y}}_g={N}_g^{-1}{\sum}_{i=1}^{N_g}{Y}_{ig}$ and ${\overline{X}}_g={N}_g^{-1}{\sum}_{i=1}^{N_g}{X}_{ig}$. $\left({\overline{X}}_A-{\overline{X}}_B\right){\hat{\beta}}_A$ indicates the difference in the outcome variable between the two groups due to differences in observable characteristics. ${\overline{X}}_B\left({\hat{\beta}}_A-{\hat{\beta}}_B\right)$ displays the difference due to the difference in coefficients.

Given that the decomposition developed in Eq. (3) is not appropriate for non-linear (NL) models, the conditional expectations *E*(*Y*_*ig*_| *X*_*ig*_) may not be the same from ${\overline{X}}_g{\hat{\beta}}_g$. Redefining in a general version the eq. (3) in terms of conditional expectations give the following equation:

4 $$ {\Delta }_A^{NL}=\left[{\mathrm{E}}_{\beta_A}\left({Y}_{iA}|{X}_{iA}\right)-{E}_{\beta_A}\left({Y}_{iB}|{X}_{iB}\right)\right]+\left[{E}_{\beta_A}\left({Y}_{iB}|{X}_{iB}\right)-{E}_{\beta_B}\left({Y}_{iB}|{X}_{iB}\right)\right] $$

Where, ${E}_{\beta_g}\left({Y}_{ig}|{X}_{ig}\right)$ represents to the conditional expectation of *Y*_*ig*_, and ${E}_{\beta_g}\left({Y}_{ih}|{X}_{ih}\right)$ stands for the conditional expectation of *Y*_*ih*_ evaluated at the parameter vector *β*_*g*_, with *g*, *h* = (*A*, *B*) and *g* ≠ *h*. When changing the reference group, an alternative expression for the decomposition is given by:

5 $$ {\Delta }_B^{NL}=\left[{E}_{\beta_B}\left({Y}_{iA}|{X}_{iA}\right)-{E}_B\left({Y}_{iB}|{X}_{iB}\right)\right]+\left[{E}_{\beta_A}\left({Y}_{iA}|{X}_{iA}\right)-{E}_{\beta_B}\left({Y}_{iA}|{X}_{iA}\right)\right] $$

The first term of the right-hand side displays the part of the differential in the dependent variable between the two groups due to the difference in explanatory variables *X*_*ig*_, and the second term displays the part of the differential in *Y*_*ig*_ due to the differences in coefficients.

The non-linear decomposition model can then present the same issues of the Oaxaca-Blinder decomposition, particularly the potential sensitivity of results. The extension of the baseline model provides the following decomposition of the Oaxaca-Blinder Decomposition:

6 $$ {\overline{Y}}_A-{\overline{Y}}_B=\left({\overline{X}}_A-{\overline{X}}_B\right){\beta}_B+{\overline{X}}_B\left({\beta}_A-{\beta}_B\right)+\left({\overline{X}}_A-{\overline{X}}_B\right)\left({\beta}_A-{\beta}_B\right)=E+C+ CE $$

Where, *E* represents the part related to the raw differential due to differences in endowments, *C* stands for the portion attributable to differences in coefficients, and *CE* reflects the part that can be explained by the interaction between *C* and *E* components. These three components can be expressed through the general version of the decomposition:

7 $$ E=\left[{E}_{\beta_B}\left({Y}_{iA}|{X}_{iA}\right)-{E}_{\beta_B}\left({Y}_{iB}|{X}_{iB}\right)\right] $$

8 $$ C=\left[{E}_{\beta_A}\left({Y}_{iB}|{X}_{iB}\right)-{E}_{\beta_B}\left({Y}_{iB}|{X}_{iB}\right)\right] $$

9 $$ CE=\left[{E}_{\beta_A}\left({Y}_{iA}|{X}_{iA}\right)-{E}_{\beta_B}\left({Y}_{iA}|{X}_{iA}\right)\right]+\left[{E}_{\beta_A}\left({Y}_{iB}|{X}_{iB}\right)-{E}_{\beta_B}\left({Y}_{iB}|{X}_{iB}\right)\right] $$

Finally, the Eq. (6) can be estimated using the sample counterparts $S\left({\hat{\beta}}_g|{X}_{ig}\right)$ and $S\left({\hat{\beta}}_h|{X}_{ig}\right)$ of the conditional expectations ${E}_{\beta_g}\left({Y}_{ig}|{X}_{ig}\right)$ and ${E}_{\beta_h}\left({Y}_{ig}|{X}_{ig}\right)$ for *g*, *h* = (*A*, *B*) and *g* ≠ *h*.

Our study aims to introduce this approach in explaining the gap in the benefit of external economic support between two groups: households with HIV/AIDS and those without HIV/AIDS. This analysis presents an extension of the Oaxaca-Blinder decomposition to a non-linear model (with limited dependent variables).

Additionally, this paper also investigated spatial (geographical) inequality in external economic support and the spread of HIV/AIDS across 31 regions of the country.

All statistical and econometrics analyses were performed with STATA SE-64 Statistical software 14.2 (StataCorp. LP, College Station, TX, USA). Spatial inequalities analysis were performed in the software R version 3.6.2.

## Results

### Descriptive statistics

Our study sample comprised 12,008 households. Table 1 presents details of households’ characteristics (heads) for the entire population and those who received economic support and those who did not receive economic support.

**Table 1:** Characteristics of the study population

Variables	Entire population (*N* = 12,008)	Received economic support (*N* = 1309)	Did not receive economic support (*N* = 10,699)	Test of independence *p*-value
HIV (%)				
HIV negative	84	83	84	0.01
HIV positive	7	9	7	
Not tested or no definite outcome	9	8	9	
Marital status (%)				
Married	59	46	61	0.00
Living together	15	10	15	
Widowed	12	27	11	
Divorced or Separated	14	17	13	
Education (%)				
No education	20	32	19	0.00
Primary	65	59	65	0.00
Secondary	14	9	15	
More than secondary	1	0	1	
External economic support (%)	11	–	–	–
Cash transfer	1	–	–	–
Assistance with school fees	1	–	–	–
Material support for education	1	–	–	–
Wealth (mean)	−0.08	−0.43	− 0.04	0.00 ^a^
Q1 (%)	23	36	21	
Q2 (%)	22	26	22	0.00
Q3 (%)	23	21	23	
Q4 (%)	18	11	19	
Q5 (%)	14	6	15	
Gender (%)				
Male	72	56	74	0.00
Female	28	44	26	
Area of residence (%)				
Rural	32	26	32	0.00
Urban	68	74	68	
Number of children (mean)	2.5	2.3	2.5	0.014 ^a^
Age – mean (SD)	45 (15)	53 (16)	44 (15)	0.00 ^a^

Note: ^a^ stand as the p-value of the Student test. SD: Standard Deviation. Source: Authors calculation based on Tanzania HIV Impact Survey 2016–2017 (THIS) - 2016-2017

Reading: The table presents the bivariate statistical test per variable, the proportion of households per subgroup (entire population; received economic support; and did not receive economic support). The sum of percentage per variable and subgroup is equal to 100%. The last column represents a bivariate test (p-value) among each variable and economic support. “*p*-value” of the chi2 test and test of difference of mean (between groups) indicated whether both variables were associated or not. However, multivariate analysis (logit model) is more robust to confirm this association

As indicated in Table 1, of the 12,008 heads of households, almost 84% of households were not infected by HIV/AIDS, 7% were infected, and 9% did not know their HIV/AIDS status. Almost 59% of households were married, 12% were widowed, and 14% were divorced or separated. Only 20% of households were not educated, 65% had attained primary education, 14% had secondary education, and only more than 1 % had a level greater than secondary. Almost 11% of the households received economic support. The wealth index quintile indicated that 23% of households were in the first quintile, 22% were in the second quintile, 23% were in the third quintile, 18% were in the fourth quintile, and 14% were in the fifth quintile. The household heads were primarily men (72%). Most households were based in rural areas (68%). The mean number of children was 2.5 per family. The mean age was 45 years (SD ± 15) with a range of 16–80.

### Econometric analysis

Table 2 presents the results of the econometric model. The logit model shows that the majority of explanatory variables were significantly associated with external economic support. Our finding indicated that being a household head living with HIV/AIDS increased the probability to receive external economic support (*p* < 0.05). The wealthier the household, the less external economic support (*p* < 0.01). Female-headed households were likely to receive external economic support (*p* < 0.01). Households in the urban areas were likely to receive less economic support than those in the rural area (*p* < 0.01). Being widowed, divorced or separated increases the probability of receiving economic support (*p* < 0.05). Age was positively associated with economic support (*p* < 0.01). However, no association was found between economic support with variables such as the number of children and education.

**Table 2:** Logit model - Factors associated with economic support in Tanzania 2016–2017

Variables	Model 1	Model 2	Model 3	Model 4	Model 5
	Economic support	Economic support	Cash transfer	Assistance with school fees	Material support for education
HIV positive	0.209*	0.320***	0.182	0.704*	0.441
	(0.107)	(0.110)	(0.348)	(0.370)	(0.338)
Wealth index	−0.629***	−0.817***	−0.567***	−0.470**	−0.349**
	(0.053)	(0.059)	(0.177)	(0.235)	(0.159)
Female	0.384***	0.386***	0.380	0.708*	0.401
	(0.090)	(0.091)	(0.307)	(0.403)	(0.304)
Urban area	−0.415***	− 0.467***	− 0.781***	− 0.567	− 0.290
	(0.089)	(0.093)	(0.272)	(0.368)	(0.274)
Number of children	− 0.017	0.005	0.056	0.148***	0.208***
	(0.014)	(0.015)	(0.046)	(0.051)	(0.037)
Education – *(No education)*	*(ref.)*	*(ref.)*	*(ref.)*	*(ref.)*	*(ref.)*
Primary	−0.025	0.049	0.477*	0.112	0.745**
	(0.074)	(0.077)	(0.264)	(0.339)	(0.290)
Secondary	0.204	0.143	0.938**	0.214	1.034***
	(0.129)	(0.133)	(0.420)	(0.590)	(0.392)
More than secondary	−0.800	−0.665	2.233***	NE	NE
	(0.724)	(0.727)	(0.833)	NE	NE
Marital status – *(Married)*	*(ref.)*	*(ref.)*	*(ref.)*	*(ref.)*	*(ref.)*
Living together	−0.185*	− 0.078	0.137	−0.417	− 0.239
	(0.104)	(0.106)	(0.344)	(0.545)	(0.367)
Widowed	0.373***	0.436***	0.439	0.263	0.320
	(0.109)	(0.111)	(0.365)	(0.486)	(0.374)
Divorced or Separated	0.229**	0.278***	0.494	0.512	0.267
	(0.104)	(0.106)	(0.349)	(0.450)	(0.366)
Age	1.455***	1.411***	1.975***	0.824*	0.852**
	(0.106)	(0.109)	(0.380)	(0.492)	(0.359)
Constant	−7.308***	−6.859***	−11.719***	−7.864***	−9.149***
	(0.453)	(0.494)	(1.702)	(2.145)	(1.678)
Number of observation	12,008	12,008	12,008	12,008	12,008
Pseudo r-squared	0.089	0.122	0.081	0.070	0.092
Chi-square	578.74	816.77	95.23	48.85	111.63
Akaike crit. (AIC)	7564.9	7347.36	1164.52	709.3	1168.0
Region effect	No	Yes	Yes	Yes	Yes
Mean dependent var	0.109	0.109	0.01	0.01	0.01
SD dependent var	0.312	0.312	0.09	0.07	0.103
Prob > chi2	0.000	0.000	0.000	0.022	0.000
AUC	0.71	0.75	0.76	0.76	0.77
HL GOF p-value^a^	0.216	0.47	0.78	0.88	0.80

Note: Standard errors in parentheses; * *p* < 0.1, ** *p* < 0.05, *** *p* < 0.01; Source: Authors calculation based on Tanzania HIV Impact Survey 2016–2017 (THIS) - 2016-2017

Reading: Model 1 is the econometric specifications of economic support, including all explanatory variables except the region effect. Additionally, model 2 included the variable related to the geographical area (region effect) as a control variable. In models 3, 4 and 5, different components of economic support were estimated as dependent variables, respectively, cash transfer, assistance for school fees, material support for education

The AUC represents the classification performance of households with economic support and those without economic support. When the AUC is near “1”, the model has a good measure of separability and “0” for a poor model meaning that it does not have a good measure of separability. “NE” stands for “not estimated” due to the lack of statistical power

^a^The Hosmer-Lemeshow goodness of fit test represents the quality of the model’s fitness with the *p*-value > 0.05; the model fits reasonably well on the validation sample

### The Oaxaca-Blinder decomposition

Table 3 provides results of the Oaxaca-Blinder decomposition of households that benefited from economic support. The results show that the difference in economic support between the two groups (HIV and no-HIV) was - 0.032 (3.2%) (*p* < 0.05). This gap was observed to favour households affected by HIV/AIDS. The results of the Oaxaca-Blinder decomposition showed that 72% (− 0.023/− 0.032) of this difference was related to the explained portion (*p* < 0.05). The wealth index (*p* < 0.01), residence area (urban) (*p* < 0.01), marital status (widowed (*p* < 0.05) and divorced or separated) (*p* < 0.1) and age (*p* < 0.01) were the essential characteristics underlying this difference between the two groups.

**Table 3:** Oaxaca-Blinder Decomposition Analysis - Factors explaining the gap in economic support in Tanzania 2016–2017

Economic support	Coef.	Std.Err.	z	P > z	[95%Conf.	Interval]	Sig
Group_1 (without HIV)	0.107	0.003	37.720	0.000	0.101	0.112	***
Group_2 (with HIV)	0.139	0.011	12.520	0.000	- 0.117	0.161	***
Difference	−0.032	0.011	−2.820	0.005	−0.055	− 0.010	***
Endowments	−0.023	0.007	−3.390	0.001	− 0.036	− 0.010	***
Coefficients	−0.023	0.012	−1.980	0.048	−0.046	−0.000	**
Interaction	0.014	0.007	1.950	0.051	−0.000	0.028	*
Endowments (E) – *Explained*							
Wealth	0.002	0.001	3.980	0.000	0.001	0.004	***
Female	−0.007	0.005	−1.440	0.150	−0.017	0.003	
Urban area	−0.005	0.002	−2.630	0.009	−0.009	−0.001	***
Number of children	0.001	0.002	0.340	0.737	−0.003	0.004	
Education==Primary	−0.001	0.001	−0.690	0.488	−0.003	0.002	
Education==Secondary	0.001	0.002	0.710	0.480	−0.002	0.004	
Education==More than secondary	0.000	0.000	0.000	0.000	0.000	0.000	
Marital==Living together	0.000	0.000	0.310	0.757	−0.001	0.001	
Marital==Widowed	−0.008	0.004	−1.970	0.049	−0.017	−0.000	**
Marital==Divorced or Separated	−0.005	0.003	−1.730	0.084	−0.011	0.001	*
Age	−0.001	0.000	−5.750	0.000	−0.001	−0.000	***
Coefficients (C) – *Unexplained*							
Wealth	−0.003	0.005	−0.690	0.489	−0.013	0.006	
Female	−0.005	0.041	−0.130	0.900	−0.085	0.075	
Urban area	0.196	0.252	0.780	0.437	−0.299	0.691	
Number of children	−0.020	0.036	−0.560	0.576	−0.092	0.051	
Education==Primary	−0.041	0.066	−0.610	0.539	−0.171	0.089	
Education==Secondary	−0.004	0.013	−0.300	0.765	−0.030	0.022	
Education==More than secondary	0.000	0.000	0.000	0.000	0.000	0.000	
Marital==Living together	−0.011	0.018	− 0.610	0.545	− 0.047	0.025	
Marital==Widowed	−0.033	0.045	−0.740	0.462	−0.120	0.055	
Marital==Divorced or Separated	−0.028	0.039	−0.710	0.475	−0.104	0.049	
Age	−0.208	0.546	−0.380	0.703	−1.278	0.862	
Constant	0.134	0.543	0.250	0.805	−0.931	1.199	
Interaction (CE)							
Wealth	−0.001	0.001	−1.460	0.144	−0.003	0.000	
Female	0.001	0.007	0.130	0.897	−0.013	0.015	
Urban area	0.004	0.003	1.500	0.135	−0.001	0.010	
Number of children	−0.002	0.003	−0.690	0.492	−0.007	0.003	
Education==Primary	0.001	0.002	0.830	0.406	−0.002	0.005	
Education==Secondary	−0.001	0.002	−0.310	0.754	−0.006	0.004	
Education==More than secondary	−0.001	0.001	−1.030	0.301	−0.003	0.001	
Marital==Living together	−0.000	0.000	−0.790	0.430	−0.001	0.001	
Marital==Widowed	0.007	0.006	1.200	0.230	−0.005	0.019	
Marital==Divorced or Separated	0.005	0.004	1.200	0.232	−0.003	0.013	
Age	0.000	0.000	0.530	0.596	−0.000	0.001	

Note: Standard errors in parentheses; * *p* < 0.1, ** *p* < 0.05, *** *p* < 0.01

Source: Authors calculation based on Tanzania HIV Impact Survey 2016–2017 (THIS) - 2016-2017

### Spatial repartition of economic support and HIV/AIDS

**Fig. 1:**
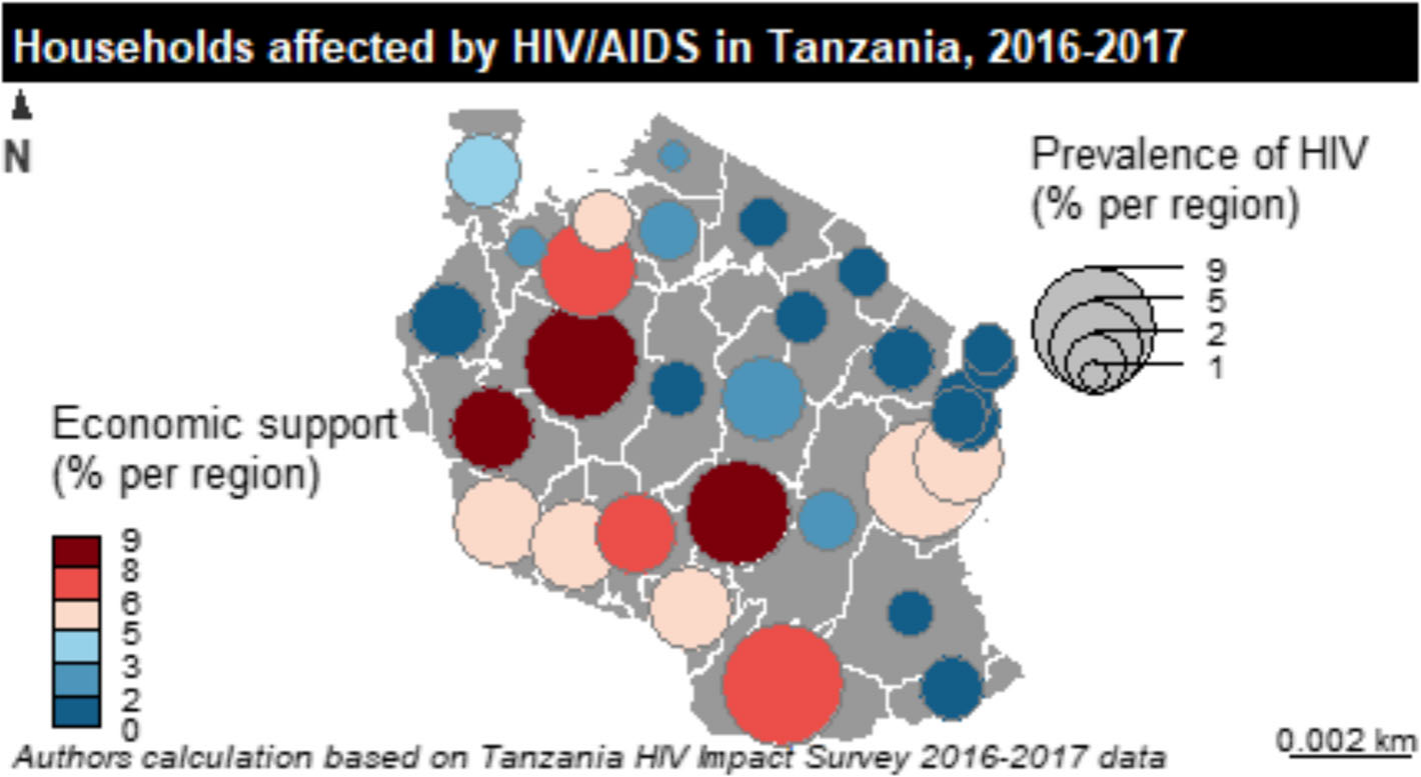
Geographical repartition of the economic support and the HIV/AIDS prevalence per region in Tanzania

Figure 1 presents a mapping of economic support and households living with HIV/AIDS across 31 regions in Tanzania. Findings show that some households living with HIV/AIDS received economic support, particularly Iringa, Ruvuma and Tabora. However, most heads of households living with HIV/AIDS in the Katavi and Mbaya regions did not receive economic support.

## Discussion

This paper disentangles the inequality of external economic support for households affected by HIV/AIDS in Tanzania regarding the lack of empirical studies in sub-Saharan African countries. Based on the results, the observed gap (72%) was likely to be associated with the explained portion, which implies that this gap may not exist if the explained variability in the group of a household affected by HIV/AIDS becomes the same in the group not affected. According to our results, the main socio-demographic characteristics explaining this difference across households affected or not by HIV/AIDS were wealth (level of poverty), residence area, marital status and age.

This study is the first to perform the Oaxaca-blinder decomposition to account for group differences in the effect of determinants (socioeconomic characteristic) given by the portion explained or endowment, as well as the difference in the effect of the determinants (portion unexplained or coefficient) for households heads living or not with the HIV/AIDS. This interesting approach reveals the cause of the regional inequalities and assists in designing an effective intervention program for economic support.

Our results indicated that less wealthy households were more likely to receive economic support according to the wealth index. This result was confirmed by the decomposition of the difference in external economic support. It appears a small proportion of less wealthy households were more concerned with the strategic support plan designed to improve the quality of life, health and reduce poverty among the vulnerable population. However, not all households (almost 11%) benefited from economic support, given the burden and socioeconomic consequences of HIV/AIDS for families [31, 39]. The results can also be explained because poor households may have low HIV/AIDS-related knowledge [40, 41].

The external economic support varied according to the living area. Households based in the urban area were less likely to receive support. Although intuitive, this result could explain the difference in economic support among population groups because the rural area disclosure is also high for most households [42]. Subsequently, households in rural areas affected by the illness would be more impacted by financial/material hardship and the disease’s burden than those in the urban area [43–47].

Given that marital status is associated with the disclosure of HIV/AIDS [42], the results show that cohabitation may reduce external economic support. This finding could be because, for married people, it is assumed that the economic standing of the couple/family will be more resilient to external shocks and the burden of the disease. Therefore, the pooling of resources at the household level for married people may reduce the illness’s shocks and strain. However, living alone, separated or divorced may significantly increase the probability of requiring external economic support. When comparing both situations, household heads living alone may experience more financial or material hardship from the illness because they cannot rely on family members; once again, they have to find a solution by themselves. They should be more concerned about external economic support. According to the literature and assuming socioeconomic consequences of HIV/AIDS and the existing barriers to health services utilisation, there is a mitigating effect of the economic support to lower the financial/material burden of the illness. The outcome of external economic support granted to households with HIV/AIDS varies across the globe and is on the Global Fund’s agenda [48] but remains low (almost 11%) in Tanzania based on our analysis. In a broad sense, this is problematic because health financing for some chronic diseases in the low-and middle-income countries shows a declining trend in international donor aid for non-communicable diseases, particularly over the past decade [49]. However, international assistance has precisely focused on communicable diseases (infant mortality rate, HIV/AIDS, malaria and tuberculosis) in Tanzania. Around 40% of the tuberculosis fund was funded by international sources [50].

The age analysis shows that the benefit from external economic support increases with age among heads of households living with HIV/AIDS. Nevertheless, HIV/AIDS is difficult to manage for old-aged persons. The old-aged in developing countries are sometimes abandoned and do not always have suitable life conditions compared to developed countries with a well-organised framework for care management. For instance, old-aged persons are exposed to poverty as they can use their limited income for care and/or pay the hospital costs of their children infected by the virus [51].

This study has several strengths and limitations that should be mentioned. On the one hand, this work is based on recent nationally representative data set. Secondly, our study was the first of this kind in Tanzania and employed further analytical procedures regarding economic support. Conversely, there is no denying that secondary data can present less control about the data and the fact that the purpose of the survey data collection was not the same as this paper’s aim. Also, the variable related to the serological status (positive/negative) could be biased when considering households that did not have the test or a definite outcome during the data collection.

Additionally, our study assumed that respondent was likely to receive at least one type of external economic support. We did not consider all the different forms of economic support separately, mainly due to the quality and nature of data and how households’ heads answered the survey. However, models with single economic support components may suffer from a lack of statistical power due to the respondent’s small sample and should be interpreted with caution.

## Conclusion

This paper investigated the difference in external economic support among households affected by HIV/AIDS (or otherwise) using the Tanzania HIV Impact Survey 2016–2017 (*THIS*) data set. The results suggest that socio-demographic characteristics such as wealth, residence area, marital status, and age explained this difference across households affected or not by HIV/AIDS. These findings have important health policy implications regarding future economic support strategies for HIV/AIDS affected households. First, promoting the prevention of HIV/AIDS campaigns to encourage households (including children) to know their serological status will be necessary for earlier care that could help in preventing financial burden and reducing inequality among people. Second, there is a need to implement a multi-year strategic plan at the community level to spread external economic support for low-income households and regions better. Finally, large-scale studies are required to assess the cost-effectiveness of different economic support strategies for HIV/AIDS affected households.

## Data Availability

The datasets supporting the conclusions of this article are available in the Population-Based HIV Impact Assessment (PHIA) project’s repository, https://phia-data.icap.columbia.edu/files#tanzania.

## Abbreviations

AIDS: Acquired immunodeficiency syndrome
HIV: Human Immunodeficiency Virus
THIS: Tanzania HIV Impact Survey
PHIA: Population-based HIV Impact Assessment

## Footnotes

1 Behaviours associated with HIV acquisition and transmission, HIV comorbidities, and other health condition were also collected. The reference “90–90-90” set by UNAIDS to control the HIV epidemic by 2020 was used.

2 The survey was conducted on 14,811 households. Within the surveyed households, eligible persons were 36,087 adults aged 15 years and above and 10,452 children aged 0–14 years (7477 children aged 0–9 years as well as 2975 early adolescents aged 10–14 years).
